# Reduction of lung metastasis, cell invasion, and adhesion in mouse melanoma by statin-induced blockade of the Rho/Rho-associated coiled-coil-containing protein kinase pathway

**DOI:** 10.1186/1756-9966-29-127

**Published:** 2010-09-16

**Authors:** Yasuhiro Kidera, Masanobu Tsubaki, Yuzuru Yamazoe, Kaori Shoji, Haruyuki Nakamura, Mitsuhiko Ogaki, Takao Satou, Tatsuki Itoh, Misako Isozaki, Junichi Kaneko, Yoshihiro Tanimori, Masashi Yanae, Shozo Nishida

**Affiliations:** 1 Division of Pharmacotherapy, Kinki University School of Pharmacy, Kowakae, Higashi-Osaka 577-8502, Japan; 2 Department of Pharmacy, Kinki University Hospital, Osakasayama, Osaka 589-8511, Japan; 3 Department of Pharmacy, Higahiosaka City General Hospital, Higashi-osaka, Osaka 578-8588, Japan; 4 Department of Pathology, Kinki University School of Medicine, Osakasayama, Osaka 589-8511, Japan; 5 Department of Pharmacy, Sakai Hospital, Kinki University School of Medicine, Sakai, Osaka 590-0132, Japan

## Abstract

**Background:**

Melanomas are highly malignant and have high metastatic potential; hence, there is a need for new therapeutic strategies to prevent cell metastasis. In the present study, we investigated whether statins inhibit tumor cell migration, invasion, adhesion, and metastasis in the B16BL6 mouse melanoma cell line.

**Methods:**

The cytotoxicity of statins toward the B16BL6 cells were evaluated using a cell viability assay. As an experimental model, B16BL6 cells were intravenously injected into C57BL/6 mice. Cell migration and invasion were assessed using Boyden chamber assays. Cell adhesion analysis was performed using type I collagen-, type IV collagen-, fibronectin-, and laminin-coated plates. The mRNA levels, enzyme activities and protein levels of matrix metalloproteinases (MMPs) were determined using RT-PCR, activity assay kits, and Western blot analysis, respectively; the mRNA and protein levels of vary late antigens (VLAs) were also determined. The effects of statins on signal transduction molecules were determined by western blot analyses.

**Results:**

We found that statins significantly inhibited lung metastasis, cell migration, invasion, and adhesion at concentrations that did not have cytotoxic effects on B16BL6 cells. Statins also inhibited the mRNA expressions and enzymatic activities of matrix metalloproteinases (MMPs). Moreover, they suppressed the mRNA and protein expressions of integrin α_2_, integrin α_4_, and integrin α_5 _and decreased the membrane localization of Rho, and phosphorylated LIM kinase (LIMK) and myosin light chain (MLC).

**Conclusions:**

The results indicated that statins suppressed the Rho/Rho-associated coiled-coil-containing protein kinase (ROCK) pathways, thereby inhibiting B16BL6 cell migration, invasion, adhesion, and metastasis. Furthermore, they markedly inhibited clinically evident metastasis. Thus, these findings suggest that statins have potential clinical applications for the treatment of tumor cell metastasis.

## Background

Metastatic melanoma is a highly aggressive, often fatal malignancy, which exhibits resistance to all the current therapeutic approaches. At the time of diagnosis, about 20% of melanoma patients already have metastatic disease. Once metastasis has occurred, the overall median survival is only 6-9 months [1]. The recent increase in the incidence of melanoma has brought to light the need for novel molecular approaches for treating melanoma metastasis [2].

Metastasis is a complex process that is dependent on the capacity of cancer cells to invade and migrate into adjoining cells and tissues, and proliferate into tumor growths [3,4]. Consistent with this definition, cell invasion and migration are highly related to the activity of matrix metalloproteinases (MMPs) that regulate many processes involved in tumor evolution, such as cell growth, migration, and extracellular matrix (ECM) degradation [5]. Notably, MMP-1, MMP-2, MMP-9, and MMP-14 (MT1-MMP) have been implicated in the invasion and metastatic processes in several cancers [6,7].

Cell adhesion is an essential process of metastatic cascades. Integrin-mediated cell adhesion affects the formation of focal adhesions, which are multimolecular structures that enable firm adhesion of cells. Integrins are a family of heterodimeric cell-surface adhesion receptors composed of α and β subunits [8,9]. Each integrin binds specific ECM components to aggregates present in the cell membrane. Changes in the structure and/or expression of integrins are frequently associated with malignant transformation and tumor progression [8,10]. It has been reported that in highly metastatic melanomas, the expression of ECM receptors such as α_2_β_1 _integrin, α_3_β_1 _integrin and α_4_β_1 _integrin is generally up-regulated [11,12].

The mevalonate metabolic pathway is essential for membrane formation and the isoprenylation of a number of small GTPases, which are involved in cell growth and differentiation. The products of this pathway include farnesyl pyrophosphate and geranylgeranyl pyrophosphate, which modify and direct small GTPases to their site of action [13,14]. The protein targets for isoprenylation include small G proteins, which require post-translational modification to undergo a series of changes that lead to their attachment to the plasma membranes and make them fully functional. The farnesylated Ras proteins are associated with the mitogenic signal transduction that occurs in response to growth factor stimulation [15]. The geranylgeranylated proteins of the Rho family include RhoA, Rac1, and Cdc42; these proteins regulate signal transduction from receptors in the membrane in a variety of cellular events related to cell adhesion to the ECM, cell morphology, cell motility, and invasion, thereby acting as molecular switches in the cell [16].

3-hydroxy-3-methylglutaryl-coenzyme A (HMG-CoA) reductase is considered to be the major regulatory enzyme of mevalonate metabolic pathway. HMG-CoA reductase inhibitors (statins) are reversible inhibitors of the rate-limiting step in cholesterol biosynthesis [17]. Most experimental studies using statins have focused on the effects of drugs on tumor cell growth *in vitro *and *in vivo *[18-21]. However, limited information is available on the effects of these agents on tumor cell invasion, adhesion, and metastasis [22-25]. Furthermore, there are no detailed reports on the exact mechanism of the inhibitory effects of statins on invasion, adhesion, and metastasis of tumor cells. Statins are widely used clinically; therefore, if they are found to inhibit tumor metastasis, they could have potential use in the future. In the present study, we have investigated the mechanisms by which statins inhibit tumor cell migration, invasion, adhesion, and metastasis in the mouse melanoma cell line B16BL6.

## Materials and methods

### Materials

Simvastatin was purchased from Wako (Osaka, Japan), and fluvastatin was purchased from Calbiochem (San Diego, CA, USA). These reagents were dissolved in dimethyl sulfoxide (DMSO) and filtered through 0.45-μm syringe filters (IWAKI GLASS, Japan). The dissolved regents were resuspended in phosphate-buffered saline (PBS; pH 7.4) and used in the various assays described below.

Y27632, a Rho-associated coiled-coil-containing protein kinase (ROCK) inhibitor, was purchased from Wako and dissolved in DMSO. The dissolved regent was resuspended in PBS and filtered through syringe filters before use.

### Cell culture

B16 melanoma BL6 cells (B16BL6 cells) were supplied by Dr. Inufusa (Kinki University, Osaka, Japan) and cultured in RPMI 1640 medium (Sigma) supplemented with 10% fetal calf serum (FCS) (Gibco, Carlsbad, CA, USA), 100 μg/ml penicillin (Gibco), 100 U/ml streptomycin (Gibco), and 25 mM HEPES (pH 7.4; Wako, Tokyo, Japan) in an atmosphere containing 5% CO_2_.

### Mice

Female C57BL/6J mice (age, 8 weeks) were purchased from Shimizu Laboratory Animals (Kyoto, Japan). The mice were maintained in a pathogen-free environment at 25°C under controlled lighting (12-h light/12-h dark cycles) and allowed free access to water and food pellets. All animal studies were performed in accordance with the Recommendations for Handling of Laboratory Animals for Biomedical Research compiled by the Committee on Safety and Ethical Handling Regulations for Laboratory Animal Experiments, Kinki University. The ethical procedures followed met the requirements of the UKCCCR guidelines (1998).

### Experimental metastasis of tumor cells

B16BL6 cells (1 × 10^5 ^cells in 0.2 ml) were injected into the tail vein of syngeneic C57BL/6J mice, after viable cells were counted with trypan blue exclusion. The mice were anesthetized with pentobarbital and sacrificed at 14 d after the cell injection. Subsequently, their lungs were excised and fixed in a neutral-buffered formaldehyde solution. Nodules visible as black forms in the lungs were then enumerated.

### Effects of oral administration of statins on lung metastasis of tumor cells

B16BL6 cells (1 × 10^5 ^cells in 0.2 ml) were injected into the tail vein of syngeneic C57BL/6J mice, after viable cells were counted with trypan blue exclusion. In the experiment, the B16BL6-inoculated mice were randomly divided into 3 groups comprising 9 mice each. For 14 d from the day of inoculation, 0.1% DMSO was administered orally to the first group, which was defined as the control group, whereas simvastatin or fluvastatin (10 mg/kg/d) was administered to the remaining 2 groups.

### Cell viability

Cell viability was assessed by the tetrazolium dye procedure by using a TetraColor ONE assay kit (Seikagaku, Tokyo, Japan). B16BL6 cells (2000 cells/well) were plated in 96-well plates and incubated with 0.01, 0.05, 0.1, and 0.5 μM fluvastatin, or 0.1, 0.5, 1, and 5 μM simvastatin for 1, 3, or 5 d. The absorbance values of the wells were measured at 492 nm by using a microplate reader (SK601; Seikagaku).

### Western blotting

B16BL6 cells treated under various conditions were lysed with a lysis buffer (20 mM Tris-HCl [pH 8.0], 150 mM NaCl, 2 mM ethylenediaminetetraacetic acid [EDTA], 100 mM NaF, 1% NP-40, 1 μg/ml leupeptin, 1 μg/ml antipain, and 1 mM phenylmethyl sulfonyl fluoride [PMSF]), and the protein concentrations of the resulting cell lysates were determined using a BCA protein assay kit (Pierce, Rockford, IL, USA). The membrane fraction of B16BL6 cells was extracted using the ProteoExtract Native Membrane Protein Extraction Kit (Calbiochem). A 40-μg protein aliquot of each extract was fractionated by electrophoresis in a sodium dodecyl sulfate-polyacrylamide gel (SDS-PAGE) and transferred to a polyvinylidene fluoride (PVDF) membrane (Amersham, Arlington Heights, IL, USA). The membranes were blocked with a solution containing 3% skim milk, and then incubated overnight at 4°C with each of the following antibodies: anti-phospho-LIMK antibody, anti-LIMK antibody, anti-phospho-MLC antibody (Cell Signaling Technology, Beverly, MA, USA), anti-MMP-14 antibody (Calbiochem), anti-α_2 _integrin antibody (Chemicon Int. Inc., California, USA), anti-α_4 _integrin antibody (SantaCruz Biotechnology, CA, USA), anti-α_5 _integrin antibody (SantaCruz Biotechnology), and anti-Rho antibody (Upstate Biology, Charlottesville, VA, USA). Subsequently, the membranes were incubated for 1 h at room temperature with anti-rabbit IgG sheep antibody coupled to horseradish peroxidase (Amersham). Reactive proteins were visualized using a chemiluminescence kit (Amersham) according to the manufacturer's instructions. Mouse anti-β-actin monoclonal antibody (Sigma) was used as the primary antibody (internal standard) for detecting β-actin protein.

### Reverse transcription-polymerase chain reaction

Total RNA was isolated using TRIzol reagent (Invitrogen, Carlsbad, CA, USA), and a 1-μg aliquot of purified total RNA was subjected to reverse transcription-polymerase chain reaction (RT-PCR) analysis using a SuperScript First-Strand Synthesis System for RT-PCR (Invitrogen). The resulting cDNAs were used as a template for PCR amplification to generate products corresponding to the mRNAs encoding various gene products. Each PCR reaction mixture contained cDNA, dNTP mix (Takara Biomedical, Shiga, Japan), 10× PCR buffer (Takara Biomedical), and Pyrobest (Takara Biomedical). The cDNAs were amplified under the following cycling conditions: For GADPH, the cDNA was amplified with 30 cycles of denaturation at 94°C for 0.5 min, annealing at 60°C for 0.5 min, and extension at 72°C for 0.5 min; and for MMP-1, MMP-2, MMP-9, MMP-14, integrin α_1_, integrin α_2_, integrin α_3_, integrin α_4_, integrin α_5_, and integrin α_6_, the cDNA was amplified with 35 cycles of denaturation at 94°C for 1 min, annealing at 55°C for 1 min, and extension at 72°C for 2 min were carried out. All PCR amplifications were performed using a DNA thermal cycler (Takara PCR thermal cycler MP; Takara Biomedical). The following primers were used: MMP-1, 5'-CGA CTC TAG AAA CAC AAG AGC AAG A-'3 (5'-primer) and 5'-AAG GTT AGC TTA CTG TCA CAC GCT T-3' (3'-primer); MMP-2, 5'-TGT GTC TTC CCC TTC ACT TT-'3 (5'-primer) and 5'-GAT CTG AGC GAT GCC ATC AA-3' (3'-primer); MMP-9, 5'-AGG CCT CTA CAG AGT CTT TG-3' (5'-primer) and 5'-CAG TCC AAC AAG AAA GGA CG-3' (3'-primer); MMP-14, 5'-ACA CCC TTT GAT GGT GAA GG-3' (5'-primer) and 5'-TCG GAG GGA TCG TTA GAA TG-3' (3'-primer); integrin α_1_, 5'-CCT GTA CTG TAC CCA ATT GGA TGG-3' (5'-primer) and 5'-GTG CTC TTA TGA AAG TCG GTT TCC-3' (3'-primer); integrin α_2_, 5'-TCT GCG TGT GGA CAT CAG TTT GGA-3' (5'-primer) and 5'-GAT AAC CCC TGT CGG TAC TTC TGC-3' (3'-primer); integrin α_3_, 5'-ATT GAC TCA GAG CTG GTG GAG GAG-3' (5'-primer) and 5'-TAC TTG GGC ATA ATC CGG TAG TAG-3' (3'-primer); integrin α_4_, 5'-GTC TTC ATG CTC CCA ACA GC-3' (5'-primer) and 5'-ACT TCT GAC GTG ATT ACA GGA AGC-3' (3'-primer); integrin α_5_, 5'-CTG CAG CTG CAT TTC CGA GTC TGG-3' (5'-primer) and 5'-GAA GCC GAG CTT GTA GAG GAC GTA-3' (3'-primer); integrin α_6_, 5'-GAG GAA TAT TCC AAA CTG AAC TAC-3' (5'-primer) and 5'-GGA ATG CTG TCA TCG TAC CTA GAG-3' (3'-primer); GAPDH, 5'-ACT TTG TCA AGC TCA TTT-3' (5'-primer) and 5'-TGC AGC GAA CTT TAT TG-3' (3'-primer). The PCR products were mixed with bromophenol blue (loading buffer) and separated by electrophoresis in a 2% agarose gel in Tris-acetate-EDTA (TAE) buffer. After staining with ethidium bromide, the bands obtained after PCR were visualized under ultraviolet light and recorded with a Coolsaver (ATTO, Tokyo, Japan).

### In vitro migration and invasion assays

Migration was analyzed by Boyden chamber assays using Falcon cell culture inserts (8.0 μm pore size; Becton Dickinson, Franklin Lakes, NJ, USA). Invasive properties of the cells were analyzed using Falcon cell culture inserts covered with 50 μg of Matrigel (Becton Dickinson) per filter. Adjusted viable cells concentration was counted with trypan blue exclusion. The upper and lower chambers of the inserts for both assays were filled with 500 μl of a cell and drug suspension (1 × 10^4 ^cells) and 1 ml of NIH/3T3 fibroblast-conditioned medium, respectively. After incubation for 24 h, the remaining cells in the upper layer were swabbed with cotton, and the cells that had penetrated the lower layer were fixed with 95% ethanol and extracted for hematoxylin staining. The cells that passed through each 8-μm pore of the culture insert were counted under a light microscope.

### Collagenase activities

Type I and type IV collagenase activities were measured using the appropriate assay kits (YAGAI Corp., Yamagata, Japan). Type I or type IV collagen was briefly labeled with fluorescein isothiocyanate (FITC) and added to the reaction mixture; this was followed by subsequent incubation for 2 h at 37°C (for type I collagenase; MMP-1) or 42°C (for type IV collagenases; MMP-2 and MMP-9). The remaining substrate was precipitated with ethanol and extracted after centrifugation at 8000 rpm for 10 min. The release of denatured collagen into the supernatant of each reaction mixture was measured by the intensity of the fluorescence emission at 520 nm following excitation at 495 nm.

### Adhesion assay

For this assay, 24-well tissue culture plates were coated with collagen I, collagen IV, fibronectin, or laminin (Becton Dickinson Biosciences) and incubated at 37°C in a 5% CO_2 _atmosphere for 1 h. Immediately before use, the coated wells were overlaid with 1% bovine serum albumin (BSA) for 30 min, washed 5 times with PBS, and dried for 30 min at room temperature in the tissue culture hood. Adjusted viable cells concentration was counted with trypan blue exclusion. The cells were loaded into individual wells (1 × 10^4 ^cells/well) and incubated for 30 min at 37°C in a 5% CO_2 _atmosphere. Nonadherent cells were aspirated and washed 3 times. Adherent cells were counted under an Olympus microscope (Olympus, Tokyo, Japan) at 20× magnification. The measurements were conducted in triplicate for each experimental group.

### Statistical analysis

All the results were expressed as the mean ± SD of several independent experiment values. Multiple comparisons of the data were performed by analysis of variance (ANOVA) with Dunnett's test. *P *values < 1% were regarded as significant.

## Results

### Cytotoxicity toward B16BL6 cells

**Figure 1:**
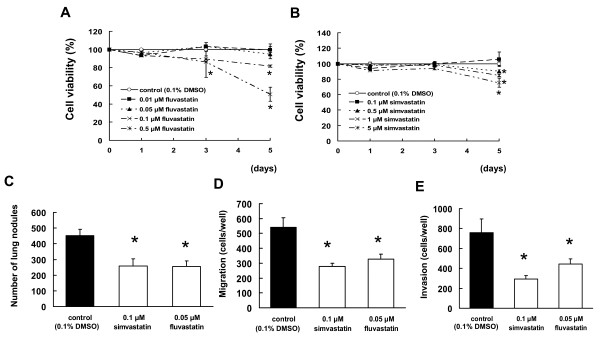
**Inhibitory effect of statins on tumor cell metastasis, migration, and invasion**. (A, B) Determination of the statin concentrations suitable for administration to B16BL6 cells. The cells were incubated in 96-well plates for 24 h and then treated with 0.01-0.5 μM fluvastatin, or 0.1-5 μM simvastatin. After 1, 3, or 5 d, cell viability was quantified by WST-8 assays. The results are representative of 5 independent experiments. (C) B16BL6 cells, which had been pretreated with 0.05 μM fluvastatin or 0.1 μM simvastatin for 3 d, were injected into the tail veins of syngeneic C57BL/6J mice. After 14 d, visible nodules that had metastasized to the lungs were counted. The results are expressed as the mean ± SD of 9 mice. (D, E) B16BL6 cells were pretreated with 0.05 μM fluvastatin or 0.1 μM simvastatin for 3 d, after which cells were seeded into the upper compartments of chambers. (D) Migration was analyzed by Boyden chamber assays using Falcon cell culture inserts. (E) Invasive properties were analyzed using Falcon cell culture inserts covered with 50 μg of Matrigel per filter. For both assays, the lower chambers contained conditioned media from NIH/3T3 cells cultured for 24 h, which was used as a chemoattractant. After incubation for 24 h, the cells invading the lower surface were counted microscopically. The results are representative of 5 independent experiments.

Cell viability of B16BL6 cells was assessed in the presence of fluvastatin (range, 0.01-0.5 μM) or simvastatin (range, 0.1-5 μM) in order to examine the cytotoxic effects of fluvastatin or simvastatin. We determined the cell survival rate, which was defined as the number of living cells as compared with the number of live control cells (0.1% DMSO-treated). The cell survival rates were calculated 1, 3, and 5 d after fluvastatin or simvastatin exposure. In the presence of 0.01, 0.05, 0.1, and 0.5 μM fluvastatin, the cell survival rates were 99.39%, 94.74%, 81.59%, and 50.77%, respectively, on day 5 (Figure 1A). In the presence of 0.1, 0.5, 1, and 5 μM simvastatin, the cell survival rates were 105.80%, 89.16%, 84.84%, and 75.52%, respectively, on day 5 (Figure 1B). A decrease in the number of B16BL6 cells was observed at day 5 after the administration of 0.1 and 0.5 μM fluvastatin or 0.5, 1, and 5 μM simvastatin (*P *< 0.01). On the basis of these results, we selected 0.05 μM and 0.1 μM as the concentrations at which fluvastatin and simvastatin, respectively, were not cytotoxic toward B16BL6 cells.

### Inhibitory effect of statins on lung metastasis in B16BL6 cells

Mice injected with tumor cells following a 3-d pretreatment with 0.05 μM fluvastatin or 0.1 μM simvastatin displayed visible lung nodules at 14 d after the injection. The numbers of pulmonary nodules following pretreatment with 0.1% DMSO (control cells), 0.1 μM simvastatin, and 0.05 μM fluvastatin were 452.6 ± 40.8, 257.6 ± 45.6, and 256.0 ± 33.9, respectively (*P *< 0.01, Figure 1C).

### Statins inhibit tumor cell migration and invasion

Cell migration and invasion are critical processes in tumor metastasis. We investigated the effects of statins on B16BL6 cell migration and invasion by the Boyden chamber and Matrigel invasion chamber assays, respectively. The number of B16BL6 cells migrating and invading through the chambers was significantly decreased by pretreatment of the cells with statins (*P *< 0.01, Figure 1D, E).

### Inhibitory effect of statins on the expressions of MMP-1, MMP-2, MMP-9, and MMP-14 in B16BL6 cells

**Figure 2:**
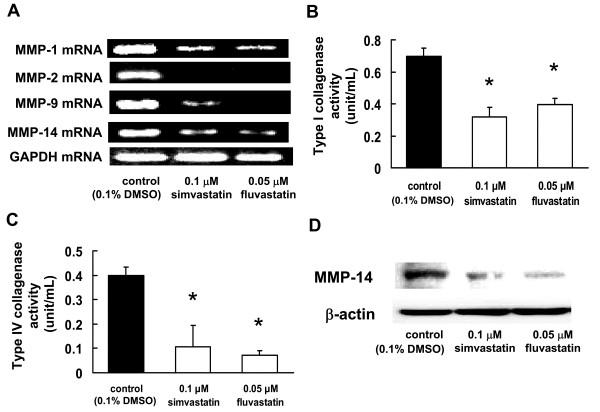
**Inhibitory effects of statins on the mRNA expressions and protein activities of MMPs**. B16BL6 cells were treated with 0.05 μM fluvastatin or 0.1 μM simvastatin for 3 d. (A) Equal amounts of total RNA were reverse-transcribed to generate cDNAs that were used for PCR analysis of the mRNA expressions of MMPs in B16BL6 cells. (B, C) Activities of (B) type I collagenase (MMP-1) and (C) type IV collagenases (MMP-2 and MMP-9) in B16BL6 cells. Conditioned media were harvested, and the type I and type IV collagenase activities were measured by FITC-conjugated type I and type IV collagen breakdown assays, respectively. The results are representative of 5 independent experiments. (D) Image showing a western blot of the MT1-MMP protein expression.

We found that statins had an inhibitory effect on invasion; this prompted us to examine its effects on the expression of MMP-1, MMP-2, MMP-9, and MMP-14. First, we examined whether statins could inhibit the expression of these MMP mRNAs. Administration of statins markedly inhibited the MMP mRNA expression of all the MMPs (Figure 2A). Next, we investigated whether type I and type IV collagenase activities and MMP-14 protein production were inhibited in B16BL6 cells that were pretreated with statins. After statins were administered, the type I and type IV collagenase activities, as well as the level of MMP-14 protein, were markedly reduced in B16BL6 cells (Figure 2B-D).

### Inhibitory effect of statins on the adhesion of B16BL6 cells to type I collagen, type IV collagen, fibronectin, and laminin

**Figure 3:**
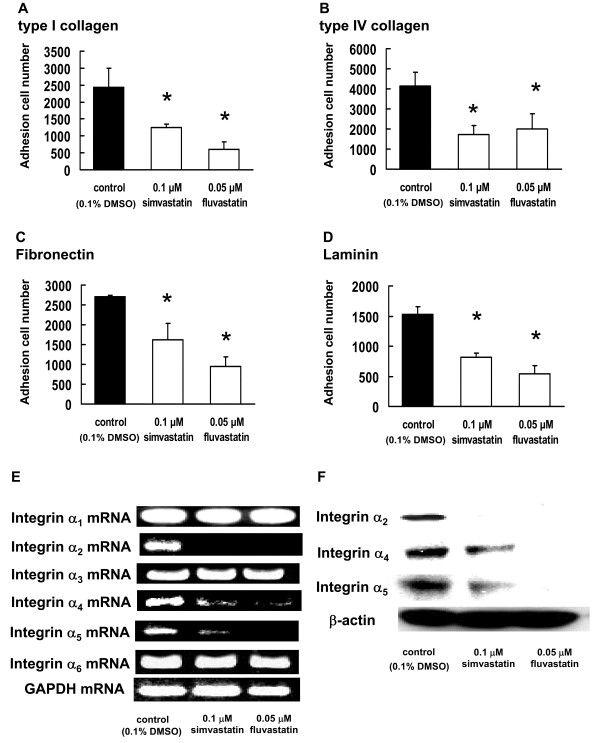
**Effect of statins on B16BL6 cell adhesion to ECM components**. B16BL6 cells, which had been treated with 0.05 μM fluvastatin or 0.1 μM simvastatin for 3 d, were incubated with (A) type I collagen-, (B) type IV collagen-, (C) fibronectin-, or (D) laminin-coated plates for 30 min at 37°C in an atmosphere containing 5% CO_2_. The results are representative of 5 independent experiments. (E) Image showing the results of RT-PCR analysis of integrins mRNA. B16BL6 cells were treated with 0.05 μM fluvastatin or 0.1 μM simvastatin. After 3 d, equal amounts of RNA were reverse-transcribed to generate cDNA, which was used for PCR analysis of integrins mRNA expression in B16BL6 cells. (E) Image showing western blot of the integrin α_2_, integrin α_4_, and integrin α_5 _proteins. Whole-cell lysates were generated and immunoblotted with antibodies against integrin α_2_, integrin α_4_, integrin α_5_, and β-actin (internal standard).

The adhesion of tumor cells to ECM components is an integrin-dependent process. The ability of tumor cells to adhere to and interact with different components of the ECM is a prerequisite for cell migration and cell invasion into the basement membrane. We investigated the effect of statins on the adhesion of B16BL6 cells to type I and type IV collagen, fibronectin, and laminin. We observed that the number of cells that adhered to type I collagen, type IV collagen, fibronectin, and laminin were significantly decreased in the presence of statins as compared to that in the 0.1% DMSO-treated cultures (control) (*P *< 0.01, Figure 3A-D).

### Suppression of integrin α_2_, integrin α_4_, and integrin α_5 _mRNA and protein expression by statins

To elucidate the effect of statins on cell adhesion to ECM components, the mRNA expression of α integrins was assessed by RT-PCR. As shown in Figure 3E, statins suppressed the mRNA expression of integrin α_2_, integrin α_4_, and integrin α_5 _in the B16BL6 cells. There was no substantial change in the level of integrin α_1_, integrin α_3_, and integrin α_6 _mRNA expressions in the statins-treated cells compared with that in the control cells (0.1% DMSO-treated). Further, we investigated whether the protein expression of integrin α_2_, integrin α_4_, and integrin α_5 _was actually inhibited in the B16BL6 cells when statins were administered; we observed that after the administration of statins, the protein expressions of integrin α_2_, integrin α_4_, and integrin α_5 _were significantly reduced (Figure 3F).

### Inhibitory effects of statins on the Rho signaling pathway

**Figure 4:**
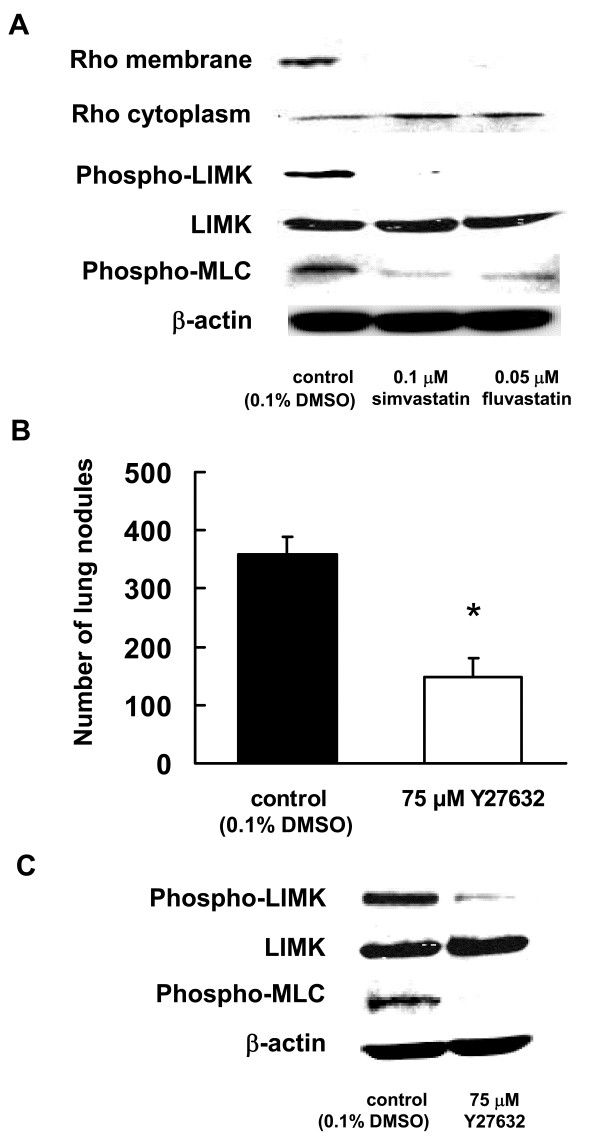
**Statins specifically suppress the Rho/ROCK pathway**. (A) B16BL6 cells were treated with 0.05 μM fluvastatin or 0.1 μM simvastatin for 3 d. Rho expression was determined by immunoblotting analysis of the membrane and cytoplasmic fractions by using the anti-Rho antibody. The expression of phosphorylated LIMK and MLC was determined by immunoblotting analysis of the whole-cell lysate using phosphorylated LIMK (phospho-LIMK) and phosphorylated MLC (phospho-MLC). (B) B16BL6 cells, which had been treated with 75 μM Y27632 for 3 d, were injected into the tail veins of syngeneic C57BL/6J mice. After 14 d, visible nodules that metastasized to the lung were counted. The results are expressed as the means ± S.D. of 9 mice. (C) B16BL6 cells were treated with 75 μM Y27632 for 3 d. The expression of phosphorylated LIMK and MLC was determined by immunoblotting analysis of the whole-cell lysate using phosphorylated LIMK (phospho-LIMK), phosphorylated MLC (phospho-MLC), and β-actin (internal standard).

To demonstrate whether statins inhibit the functions of Rho by suppressing their prenylation, the protein samples were subjected to a standard western blot assay to detect the presence of small GTPases in both the membrane and cytoplasm lysates of B16BL6 cells incubated with or without statins. The membrane localization of Rho proteins showed a significant decrease in statin-treated cells compared to the control cells (0.1% DMSO-treated). In contrast, the cytoplasmic localization of Rho proteins showed an increase in the reagent-treated cells compared to the control cells (0.1% DMSO-treated) (Figure 4A). Moreover, statins inhibited the expression of phosphorylated LIMK and MLC, as downstream of Rho. Thus, these results suggest that the Rho signaling pathway was inhibited by statins in our experiment model.

### Inhibitory effect of Y27632 on lung metastasis in B16BL6 cells

The results described so far have shown that the inhibitory effect of statins on lung metastasis is exerted via the inhibition of Rho prenylation. We next administered Y27632, a ROCK inhibitor, to B16BL6 cells in order to determine whether suppression of the Rho/ROCK pathway would cause the inhibition of lung metastasis. As observed in the case of statins, administration of Y27632 sufficiently inhibited lung metastasis (*P *< 0.01, Figure 4B). In addition, Y27632 decreased the expression of phosphorylated LIMK and MLC (Figure 4C). These results suggested that statins inhibited lung metastasis by suppressing the Rho signaling pathway.

### Inhibitory effect of oral administration of statins on tumor metastasis

**Figure 5:**
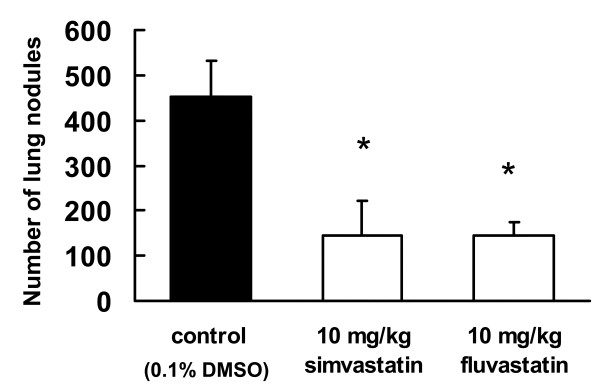
**Inhibitory effect of oral administration of statins on lung metastasis**. B16BL6 cells were injected into the tail veins of syngeneic C57BL/6J mice. Mice were treated daily from days 1 to 14 with 10 mg/kg fluvastatin or simvastatin. After 14 d, visible nodules that had metastasized to the lungs were counted. The results are expressed as the mean ± SD for 9 mice.

To determine whether oral administration of statins would inhibit metastasis, we investigated their effect on the development of metastasis in C57BL6/J mice. The results indicated that statins significantly inhibited lung metastasis (*P *< 0.01, Figure 5) when administered orally.

## Discussion

In the present study, we have demonstrated that statins inhibit cell migration, invasion, adhesion, and metastasis through the suppression of the Rho/ROCK pathway in mouse melanoma B16BL6 cells. It has been reported that overexpression of RhoA increases tumor metastasis in human melanomas [26]. It has also been reported that the overexpression of RhoC enhances metastasis, whereas dominant-negative expression of RhoC inhibits metastasis [27]. In addition, statins have been reported to inhibit tumor cell migration and invasion through the suppressing geranylgeranylation of Rho in breast and colon cancer cell lines [28,29]. These findings suggest that statins may bring about their anti-metastatic effects by inactivating the Rho/ROCK pathway.

Cell migration is known to be required for tumor metastasis. In this study, we showed that statins inhibited the migration of B16BL6 cells. It has been reported that YM529/ONO-5920 and zoledronate, nitrogen-containing bisphosphonates, inhibited hepatocellular carcinoma and osteosarcoma cell migration by suppressing GGPP biosynthesis [30,31]. Collectively, the findings suggest that the inhibition of GGPP biosynthesis plays an important role in the suppression of B16BL6 cell migration by statins.

Matrix metalloproteinases (MMPs) and zinc-dependent endopeptidases are a family of structurally related zymogens that are capable of degrading the ECM, including the basement membrane. They are presumed to be critically involved in tumor invasion and metastasis [32]. In melanomas, higher levels of MMP-1, MMP-2, MMP-9, and MMP-14 have been observed in the more invasive and metastatic tumors [33]. Moreover, overexpression of RhoA-GTP induces MMP expression and activity [34]. We observed that statins significantly inhibit the mRNA expression and enzymatic activities of MMP-1, MMP-2, MMP-9, and MMP-14 in B16BL6 cells. These results suggest that the decrease in the activation of Rho is vital for the suppression of MMP expressions by statins in B16BL6 cells.

Cell adhesion is a fundamental cellular response that is intricately involved in the physiological processes of proliferation, motility, as well as the pathology of neoplastic transformation and metastasis. Integrins are the most important family of cell surface adhesion molecules that mediate interactions between cells and the ECM. Members of the β1 integrin subfamily are known to primarily bind to collagens, fibronectins, and laminins. We found that statins suppress cell adhesion to type I collagen, type IV collagen, fibronectin, and laminin. Furthermore, statins significantly inhibited the mRNA and protein expressions of integrin α_2_, integrin α_4_, and integrin α_5_. A recent study has reported that the activation of small GTPases increased cell adhesion to collagens, fibronectins, and laminins [35]. These findings indicate that the Rho/ROCK pathway may be essential for the expressions of integrin α_2_, integrin α_4_, and integrin α_5_.

Activation of Rho could lead to the activation of LIMK and MLC [36]. These signal transduction factors are essential for cell migration, invasion, adhesion, and metastasis [37-39]. Our results clearly demonstrate that statins induced a decrease in the phosphorylation of LIMK and MLC. Moreover, we observed that Y27632, a ROCK inhibitor, inhibited tumor cell metastasis through suppressing LIMK and MLC activation. We previous reported that Y27632 suppresses tumor cell migration, invasion, and adhesion, as well as the expressions of MMPs and integrins in B16BL6 cells, and then Y27632 did not show cytotoxic effect on B16BL6 cells [40]. MMP expressions can be induced by various growth factors and cytokines, including epidermal growth factor [41]. The expression of integrins can also be induced by tumor necrosis factor alpha [42]. These inductions require the activation of the Rho pathway. Therefore, our present findings suggest that statins inhibit the expression of MMPs and integrins by suppressing the Rho/ROCK pathways.

Previous studies have shown that Rho pathway components are potential therapeutic targets for tumor progression and metastasis [43]. Farina et al. have reported that lovastatin inhibits Rho isoprenylation, migration, and metastasis in mouse mammary carcinoma cells [44]. Horiguchi et al. have also indicated that fluvastatin inhibits invasion, angiogenesis, and metastasis in renal cancers [24]. However, no detailed data have been reported on the exact mechanisms of the inhibitory effects of statins on the migration, invasion and metastasis of tumor cells. In this study, we have indicated that the inhibitory effect of statins on tumor cell migration, invasion, adhesion, and metastasis suppresses the expression of MMPs and integrins through inhibition of the Rho/ROCK pathway. These findings indicate that Rho inhibitors, such as statins, are appropriate agents for molecular therapies against malignant tumor cells.

In the present study, the treatment of B16BL6 cells with 0.05 μM fluvastatin or 0.1 μM simvastatin for 3 days in vitro. The peak plasma concentrations of fluvastatin or simvastatin achieved with standard doses were ≤1 μM or 2.7 μM, respectively [24,45]. These findings indicate that 0.05 μM and 0.1 μM of fluvastatin and simvastatin, respectively, are within the peak plasma values of fluvastatin or simvastatin that are likely to be achieved in vivo.

We also observed that statins inhibit lung metastasis when administered orally. Fluvastatin or simvastatin are usually administered orally at daily doses of 20 to 80 mg or 5 to 40 mg in patients with hypercholesterolemia. Importantly, the dosage of statins orally administered to patients with hypercholesterolemia would have prophylactic effects against metastasis. This data indicates that statins may be therapeutically useful for the treatment of a variety of tumors.

## Conclusion

In conclusion, our data show that statins inhibit tumor cell migration, invasion, adhesion, and metastasis through the suppression of the Rho/ROCK pathway. These findings suggest that statins are potentially useful as anti-metastatic agents for the treatment of melanoma.

## Competing interests

The authors declare that they have no competing interests.

## Authors' contributions

YK and MT carried out animal experiment, cell viability assay, boyden chamber assay, invasion assay, statical analysis, and drafted the manuscript. YY, KS, HN, MO, and MI carried out collagenase activity aasay, RT-PCR, and western bolotting analysis. JN participated in animal experiment. YT and MY participated in boyden chamber assay and invasion assay. TS and TI contributed to animal experiment and statistical analyses. SN designed the experiments and revised the manuscript. All authors read and approved the final manuscript.

## References

[B1] Tarhini AA Agarwala SS Cutaneous melanoma: available therapy for metastatic disease Dermatol Ther 2006 19 19 25 10.1111/j.1529-8019.2005.00052.x 16405566

[B2] Howe HL Wingo PA Thun MJ Ries LA Rosenberg HM Feigal EG Edwards BK Annual report to the nation on the status of cancer (1973 through 1998), featuring cancers with recent increasing trends J Natl Cancer Inst 2001 93 824 842 10.1093/jnci/93.11.824 11390532

[B3] Woodhouse EC Chuaqui RF Liotta LA General mechanisms of metastasis Cancer 1997 80 1529 1537 10.1002/(SICI)1097-0142(19971015)80:8+<1529::AID-CNCR2>3.0.CO;2-F 9362419

[B4] Van Noorden CJ Proteases and protease inhibitors in cancer Acta Histochem 1998 100 344 354 9842415 10.1016/s0065-1281(98)80032-9

[B5] Sternlicht MD Werb Z How matrix metalloproteinases regulate cell behavior Annu Rev Cell Dev Biol 2001 17 463 516 10.1146/annurev.cellbio.17.1.463 11687497 PMC2792593

[B6] Coussens LM Fingleton B Matrisian LM Matrix metalloproteinase inhibitors and cancer: trials and tribulations Science 2002 295 2387 2392 10.1126/science.1067100 11923519

[B7] Egeblad M Werb Z New functions for the matrix metalloproteinases in cancer progression Nat Rev Cancer 2002 2 161 174 10.1038/nrc745 11990853

[B8] Danen EH Yamada KM Fibronectin, integrins, and growth control J Cell Physiol 2001 189 1 13 10.1002/jcp.1137 11573199

[B9] Ingber DE Integrins, tensegrity, and mechanotransduction Gravit Space Biol Bull 1997 10 49 55 11540119

[B10] Chrenek MA Wong P Weaver VM Tumour-stromal interactions. Integrins and cell adhesions as modulators of mammary cell survival and transformation Breast Cancer Res 2001 3 224 229 10.1186/bcr300 11434873 PMC138686

[B11] Hartstein ME Grove AS Jr Woog JJ The role of the integrin family of adhesion molecules in the development of tumors metastatic to the orbit Ophthal Plast Reconstr Surg 1997 13 227 238 10.1097/00002341-199712000-00001 9430298

[B12] Moretti S Martini L Berti E Pinzi C Giannotti B Adhesion molecule profile and malignancy of melanocytic lesions Melanoma Res 1993 3 235 239 8219755

[B13] Grünler J Ericsson J Dallner G Branch-point reactions in the biosynthesis of cholesterol, dolichol, ubiquinone and prenylated proteins Biochim Biophys Acta 1994 1212 259 77 8199197 10.1016/0005-2760(94)90200-3

[B14] Elson CE Peffley DM Hentosh P Mo H Isoprenoid-mediated inhibition of mevalonate synthesis: potential application to cancer Proc Soc Exp Biol Med 1999 221 294 311 10.1046/j.1525-1373.1999.d01-87.x 10460692

[B15] Pronk GJ Bos JL The role of p21ras in receptor tyrosine kinase signalling Biochim Biophys Acta 1994 1198 131 147 7819271 10.1016/0304-419x(94)90010-8

[B16] Hall A Rho GTPases and the actin cytoskeleton Science 1998 279 509 514 10.1126/science.279.5350.509 9438836

[B17] Goldstein JL Brown MS Regulation of the mevalonate pathway Nature 1990 343 425 430 10.1038/343425a0 1967820

[B18] Nonaka M Uota S Saitoh Y Takahashi M Sugimoto H Amet T Arai A Miura O Yamamoto N Yamaoka S Role for protein geranylgeranylation in adult T-cell leukemia cell survival Exp Cell Res 2009 315 141 150 10.1016/j.yexcr.2008.10.010 18992741

[B19] Nishida S Matsuoka H Tsubaki M Tanimori Y Yanae M Fujii Y Iwaki M Mevastatin induces apoptosis in HL60 cells dependently on decrease in phosphorylated ERK Mol Cell Biochem 2005 269 109 114 10.1007/s11010-005-3086-0 15786722

[B20] Lu G Xiao H You H Lin Y Snagaski B Yang CS Synergistic inhibition of lung tumorigenesis by a combination of green tea polyphenols and atorvastatin Clin Cancer Res 2008 14 4981 4988 10.1158/1078-0432.CCR-07-1860 18676773

[B21] von Tresckow B von Strandmann EP Sasse S Tawadros S Engert A Hansen HP Simvastatin-dependent apoptosis in Hodgkin's lymphoma cells and growth impairment of human Hodgkin's tumors in vivo Haematologica 2007 92 682 685 10.3324/haematol.11020 17488694

[B22] Alonso DF Farina HG Skilton G Gabri MR De Lorenzo MS Gomez DE Reduction of mouse mammary tumor formation and metastasis by lovastatin, an inhibitor of the mevalonate pathway of cholesterol synthesis Breast Cancer Res Treat 1998 50 83 93 10.1023/A:1006058409974 9802623

[B23] Nübel T Dippold W Kaina B Fritz G Ionizing radiation-induced E-selectin gene expression and tumor cell adhesion is inhibited by lovastatin and all-trans retinoic acid Carcinogenesis 2004 25 1335 1344 10.1093/carcin/bgh133 14988223

[B24] Horiguchi A Sumitomo M Asakuma J Asano T Asano T Hayakawa M 3-hydroxy-3-methylglutaryl-coenzyme a reductase inhibitor, fluvastatin, as a novel agent for prophylaxis of renal cancer metastasis Clin Cancer Res 2004 10 8648 8655 10.1158/1078-0432.CCR-04-1568 15623649

[B25] Zhong WB Liang YC Wang CY Chang TC Lee WS Lovastatin suppresses invasiveness of anaplastic thyroid cancer cells by inhibiting Rho geranylgeranylation and RhoA/ROCK signaling Endocr Relat Cancer 2005 12 615 629 10.1677/erc.1.01012 16172195

[B26] Collisson EA Carranza DC Chen IY Kolodney MS Isoprenylation is necessary for the full invasive potential of RhoA overexpression in human melanoma cells J Invest Dermatol 2002 119 1172 1176 10.1046/j.1523-1747.2002.19519.x 12445208

[B27] Clark EA Golub TR Lander ES Hynes RO Genomic analysis of metastasis reveals an essential role for RhoC Nature 2000 406 532 535 10.1038/35020106 10952316

[B28] Kusama T Mukai M Tatsuta M Matsumoto Y Nakamura H Inoue M Selective inhibition of cancer cell invasion by a geranylgeranyltransferase-I inhibitor Clin Exp Metastasis 2003 20 561 567 10.1023/A:1025898316728 14598891

[B29] Kusama T Mukai M Tatsuta M Nakamura H Inoue M Inhibition of transendothelial migration and invasion of human breast cancer cells by preventing geranylgeranylation of Rho Int J Oncol 2006 29 217 223 16773203

[B30] Kogure T Ueno Y Kimura O Kondo Y Inoue J Fukushima K Iwasaki T Shimosegawa T A novel third generation bisphosphonate, minodronate (YM529), prevented proliferation and migration of hepatocellular carcinoma cells through inhibition of mevalonate pathway Hepatol Res 2009 39 479 489 10.1111/j.1872-034X.2008.00484.x 19207585

[B31] Kubista B Trieb K Sevelda F Toma C Arrich F Heffeter P Elbling L Sutterlüty H Scotlandi K Kotz R Micksche M Berger W Anticancer effects of zoledronic acid against human osteosarcoma cells J Orthop Res 2006 24 1145 1152 10.1002/jor.20129 16602111

[B32] Liotta LA Tryggvason K Garbisa S Hart I Foltz CM Shafie S Metastatic potential correlates with enzymatic degradation of basement membrane collagen Nature 1980 284 67 68 10.1038/284067a0 6243750

[B33] Hofmann UB Westphal JR Van Muijen GN Ruiter DJ Matrix metalloproteinases in human melanoma J Invest Dermatol 2000 115 337 344 10.1046/j.1523-1747.2000.00068.x 10951266

[B34] Cáceres M Guerrero J Martínez J Overexpression of RhoA-GTP induces activation of the epidermal growth factor receptor, dephosphorylation of focal adhesion kinase and increased motility in breast cancer cells Exp Cell Res 2005 309 229 238 10.1016/j.yexcr.2005.05.020 15963982

[B35] Danker K Mechai N Lucka L Reutter W Horstkorte R The small Gtpase ras is involved in growth factor-regulated expression of the alpha1 integrin subunit in PC12 cells Biol Chem 2001 382 969 972 10.1515/BC.2001.121 11501763

[B36] Hopkins AM Pineda AA Winfree LM Brown GT Laukoetter MG Nusrat A Organized migration of epithelial cells requires control of adhesion and protrusion through Rho kinase effectors Am J Physiol Gastrointest Liver Physiol 2007 292 G806 G817 10.1152/ajpgi.00333.2006 17138966

[B37] Bernard O Lim kinases, regulators of actin dynamics Int J Biochem Cell Biol 2007 39 1071 1076 10.1016/j.biocel.2006.11.011 17188549

[B38] Vega FM Ridley AJ Rho GTPases in cancer cell biology FEBS Lett 2008 582 2093 2101 10.1016/j.febslet.2008.04.039 18460342

[B39] Barkan D Kleinman H Simmons JL Asmussen H Kamaraju AK Hoenorhoff MJ Liu ZY Costes SV Cho EH Lockett S Khanna C Chambers AF Green JE Inhibition of metastatic outgrowth from single dormant tumor cells by targeting the cytoskeleton Cancer Res 2008 68 6241 6250 10.1158/0008-5472.CAN-07-6849 18676848 PMC2561279

[B40] Tanimori Y Tsubaki M Yamazoe Y Satou T Itoh T Kidera Y Yanae M Yamamoto C Kaneko J Nishida S Nitrogen-containing bisphosphonate, YM529/ONO-5920, inhibits tumor metastasis in mouse melanoma through suppression of the Rho/ROCK pathway Clin Exp Metastasis in press 10.1007/s10585-010-9342-z 20632074

[B41] Kusama T Mukai M Iwasaki T Tatsuta M Matsumoto Y Akedo H Inoue M Nakamura H 3-hydroxy-3-methylglutaryl-coenzyme a reductase inhibitors reduce human pancreatic cancer cell invasion and metastasis Gastroenterology 2002 122 308 317 10.1053/gast.2002.31093 11832446

[B42] Takemura A Nakagawa I Kawai S Inaba H Kato T Hamada S Amano A Inhibitory effects of tumor necrosis factor-alpha on migration of human periodontal ligament cells J Periodontol 2006 77 883 890 10.1902/jop.2006.050192 16671882

[B43] Chan KK Oza AM Siu LL The statins as anticancer agents Clin Cancer Res 2003 9 10 19 12538446

[B44] Farina HG Bublik DR Alonso DF Gomez DE Lovastatin alters cytoskeleton organization and inhibits experimental metastasis of mammary carcinoma cells Clin Exp Metastasis 2002 19 551 559 10.1023/A:1020355621043 12405293

[B45] Sondergaard TE Pedersen PT Andersen TL Søe K Lund T Ostergaard B Garnero P Delaisse JM Plesner T A phase II clinical trial does not show that high dose simvastatin has beneficial effect on markers of bone turnover in multiple myeloma Hematol Oncol 2009 27 17 22 10.1002/hon.869 18668701

